# Predator Personality Variation, Not the Multiple Stressors of Temperature and Light, Determines Feeding Motivation in an Ambush Piscivore *Saxatilia proteus*

**DOI:** 10.1002/ece3.70540

**Published:** 2024-11-14

**Authors:** Lucy J. Brown, Christos C. Ioannou

**Affiliations:** ^1^ School of Biological Sciences University of Bristol Bristol UK

**Keywords:** *Crenicichla*, environmental change, foraging, individual differences, multi‐stressors, pike cichlid

## Abstract

Environmental conditions in freshwater ecosystems are increasingly determined by human activity. Increased temperature and light intensity are among the anthropogenic stressors dramatically altering these ecosystems, for example, through deforestation that reduces canopy cover of riparian vegetation. Simultaneous exposure to multiple stressors complicates predictions of responses to environmental stressors due to potential interactions, yet the interaction between temperature and light intensity on feeding motivation remains poorly understood. Here, a fully factorial design was employed to investigate the combined effect of increased temperature and light intensity on the feeding motivation of a freshwater predator, the pike cichlid *Saxatilia proteus*. Strikes toward food items were used to quantify the subjects' motivation to feed. We found no effect of temperature or light intensity on feeding motivation, either individually or as an interaction. Our repeated measures design allowed us to test whether the predatory fish showed personality variation, i.e., consistent inter‐individual differences, in their motivation to feed. While the time taken to make the first strike was not consistently different between individuals, the number of strikes in 1 min, 3 min and the time taken for the 10th strike (which were strongly correlated to one another) was consistently different between individuals. This variation could not be explained by variation in body length, which had no effect as a main effect. Thus, we suggest that anthropogenic effects that alter the composition of individuals in a population of predators, for example selective harvesting, will have a greater ecological effect than direct short‐term effects of variability in environmental factors.

## Graphical abstract

Here, a fully factorial design was employed to investigate the combined effect of increased temperature and light intensity on the feeding motivation of a freshwater predator, the pike cichlid *Saxatilia proteus*. We found no effect of temperature or light intensity on feeding motivation, either individually or as an interaction. While the time taken to make the first strike was not consistently different between individuals, the number of strikes in 1 min, 3 min and the time taken for the 10th strike (which were strongly correlated to one another) was consistently different between individuals.

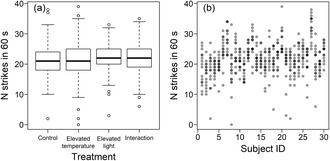

## 1 Introduction

Temperature and light are fundamental components of ecosystems and play a key role in driving species interactions by influencing foraging, and thus shaping ecological communities (Jägerbrand and Spoelstra 2023; Kordas, Harley, and O'Connor 2011). However, as human activities continue to modify ecosystems and impose novel abiotic and biotic conditions, changes to inter‐specific interactions may have complex impacts for community function and diversity (Guiden et al. 2019). Particularly in ectotherms, temperature increases energetic demand, which in turn impacts behaviour and physiology (Abram et al. 2017). Behavioural adjustments serve as the initial response to temperature fluctuations (Bailey et al. 2022), primarily manifested in changes to locomotor and feeding behaviours due to changes in the motivation to feed, thus allowing increased energy requirements to be met (Domenici et al. 2019; Volkoff and Peter 2006). While the resilience of organisms to environmental stressors is heavily species‐ and context‐dependent, tropical fish are likely to be disproportionately affected by warming because they possess narrow thermal windows and already live close to their thermal tolerance limit (Lapointe et al. 2018; Payne et al. 2016). There is already evidence that increased energy consumption, due to elevated temperatures, is intensifying top‐down competition and declining prey populations in the warming Indo‐West Pacific Ocean (Johansen et al. 2015). This phenomenon can distort entire food webs and may already be affecting vulnerable freshwater ecosystems.

For animals that rely on visual cues, light is crucial for activities including reproduction, predator avoidance and foraging (Marchesan et al. 2005). In a predator–prey context, increased light intensity can aid predators locate prey (Fleming and Bateman 2018; Richmond, Hrabik, and Mensinger 2004); conversely, it can make predators more conspicuous, inducing increased vigilance or fleeing behaviour in prey (Michels, Hrabik, and Mensinger 2024). Light in aquatic systems is predominantly influenced by surface illumination, depth and suspended particulates, which can change light intensity, colour composition and polarisation (Jägerbrand and Spoelstra 2023). Since the development of human civilisation, deforestation has reduced the planet's forests to less than 70% of their original extent (Bologna and Aquino 2020). This is significant for freshwaters because the shading provided by riparian vegetation regulates both temperature and light in freshwater habitats (Mosisch et al. 1999). In these ecosystems, which possess discrete physical boundaries, inhabitants may not be capable of large‐scale movements in response to stressors from human‐induced changes in environmental conditions (Morgan, McDonald, and Wood 2001).

Many studies have researched the isolated effects of either temperature (Alfonso, Gesto, and Sadoul 2021; Barbarossa et al. 2021) or light (Keep et al. 2021) in freshwater systems, controlling for other environmental stressors. To enhance the ecological relevance of research on responses to environmental stressors, there is an increasing focus on the effects of simultaneous exposure to multiple environmental stressors, which are ubiquitous in natural systems (Côté, Darling, and Brown 2016; Orr et al. 2020). Therefore, to form accurate predictions of their impacts it is essential to confirm how they interact as evidence suggests that they can combine in various complex ways (McFarland et al. 2012), which confounds projections of their net ecological impact (Thompson, MacLennan, and Vinebrooke 2018). Interactions between multiple human‐induced environmental stressors have been found to impose significant impacts across a range of behavioural contexts (Côté, Darling, and Brown 2016; Schmitz and Trussell 2016). Effects can be additive when the observed response is the sum of the responses from each stressor (Zanghi et al. 2024). Comparative responses are those dominated by the effect of only one stressor (Folt et al. 1999), whereas a ceiling effect may occur where the effect of an additional stressor is not evident (Ginnaw et al. 2020). An interaction between two stressors can be either synergistic (more than the sum of each response (Zanghi, Munro, and Ioannou 2023)) or antagonistic (less than the sum of each response (Ferrari et al. 2015)). Given this variability in outcomes, conducting empirical studies to assess the responses to stressors that co‐occur is vital.

In addition to variation induced by changing environmental conditions, the past few decades have seen widespread interest in consistent variation in behaviour between individuals within populations, also known as animal personality variation, that cannot be explained by other traits such as age, sex and size (Dall, Houston, and McNamara 2004; Sih et al. 2015). This research includes growing evidence that this consistent inter‐individual variation can have ecological impacts (Brehm et al. 2019; Mittelbach, Ballew, and Kjelvik 2014; Wolf and Weissing 2012). This is particularly the case regarding variation between individual predators in their motivation to feed, and hence the risk they pose to their prey (Toscano et al. 2016). Consistent variation between individual predators has been documented particularly in predators in aquatic systems, demonstrating personality variation in direct measures of feeding, including the pike cichlid *Saxatilia frenata* studied in situ (Szopa‐Comley et al. 2020), and northern pike *Esox lucius* (Nyqvist et al. 2012) and three‐spined sticklebacks *Gasterosteus aculeatus* studied in the laboratory (Szopa‐Comley, Donald, and Ioannou 2020). Additionally, similar consistent inter‐individual variation in feeding has been demonstrated in omnivorous fish such as the Nile tilapia *Oreochromis niloticus* (Silva et al. 2014). However, there were no consistent differences in predatory behaviour between individual blue acara *Andinoacara pulcher* tested under laboratory conditions (Szopa‐Comley and Ioannou 2022). Additionally, other studies have shown individual predators can consistently vary in other behaviours that are likely to covary with feeding motivation, hence the risk they pose to prey and the ecological impact of the predators. This includes activity (McLaughlin, Grant, and Kramer 1994; Nakayama et al. 2016), risk‐taking tendency, i.e., boldness (Dhellemmes et al. 2021; Krüger et al. 2019; Zhao and Feng 2015), prey search behaviour (Patrick et al. 2014; Patrick, Pinaud, and Weimerskirch 2017; Traisnel and Pichegru 2019), space use (McLaughlin, Grant, and Kramer 1994; Patrick and Weimerskirch 2014; Villegas‐Ríos et al. 2018), competitive ability (Cole and Quinn 2011), and foraging site fidelity (Harris et al. 2020). The number of attacks by individual predators has even been demonstrated to be determined by a combination of the personality traits of both the predators and the prey, as demonstrated in the spiders *Portia labiate* and *Cosmophasis umbratica* (Chang et al. 2017).

The feeding behaviour of diurnal, predatory freshwater fishes is likely to be more severely impacted by warming and changes in the light environment than species capable of range shifts or those that are nocturnal which rely less heavily on visual cues (Freitas et al. 2021). Here, we test how the combination of increased water temperature and light intensity affects the feeding motivation of a freshwater fish, *Saxatilia proteus*, a species of pike cichlid (*Crenicichla*). Pike cichlids are piscivorous fish native to South American streams, rivers and lakes, relying mainly on visual cues to ambush prey (Szopa‐Comley et al. 2020). In their natural habitat, pike cichlids will often experience elevated temperature and light intensity when changes in land use reduces canopy cover from removing riparian vegetation. Across river sites in the Northern Range mountains of Trinidad where *S. frenata* (which is closely related to *S. proteus* (Varella et al. 2023)) are abundant, Zanghi et al. (2024) demonstrated a positive correlation between temperature and light intensity, and declining light intensity with increasing canopy cover. However, temperature and canopy cover were not significantly correlated; instead, temperature was negatively associated with flow rate. In our study, strikes on food items were used to measure feeding motivation and assess the subjects' response to altered environmental conditions. Fish were exposed to control or elevated water temperature crossed with control or elevated light intensity in a fully factorial design to determine the individual and combined effects of these stressors. By using a repeated‐measures design, i.e., with repeated tests per subject over a four‐week period, we were also able to assess consistent inter‐individual variation in the feeding response of these fish.

The study of Zanghi et al. (2024) also tested for the effects of naturally occurring environmental variation on the presence and predatory behaviour of piscivore fish in their study system. The presence of *S. frenata* was associated with warmer temperatures, and across the predators included in the study, predation pressure such as the number of attacks on the guppy prey presented as a stimulus also increased with temperature. We predicted that as the effects of temperature and light on visual predators are driven by different pathways, i.e., physiological for temperature and visual for light, their effects would be additive rather than synergistic. Also based on field observations that presented live fish prey to wild pike cichlids (Szopa‐Comley et al. 2020), we expected consistent inter‐individual differences between the *S. proteus* in our study.

## 2 Methods

### 2.1 Experimental Subjects and Housing

**FIGURE 1:**
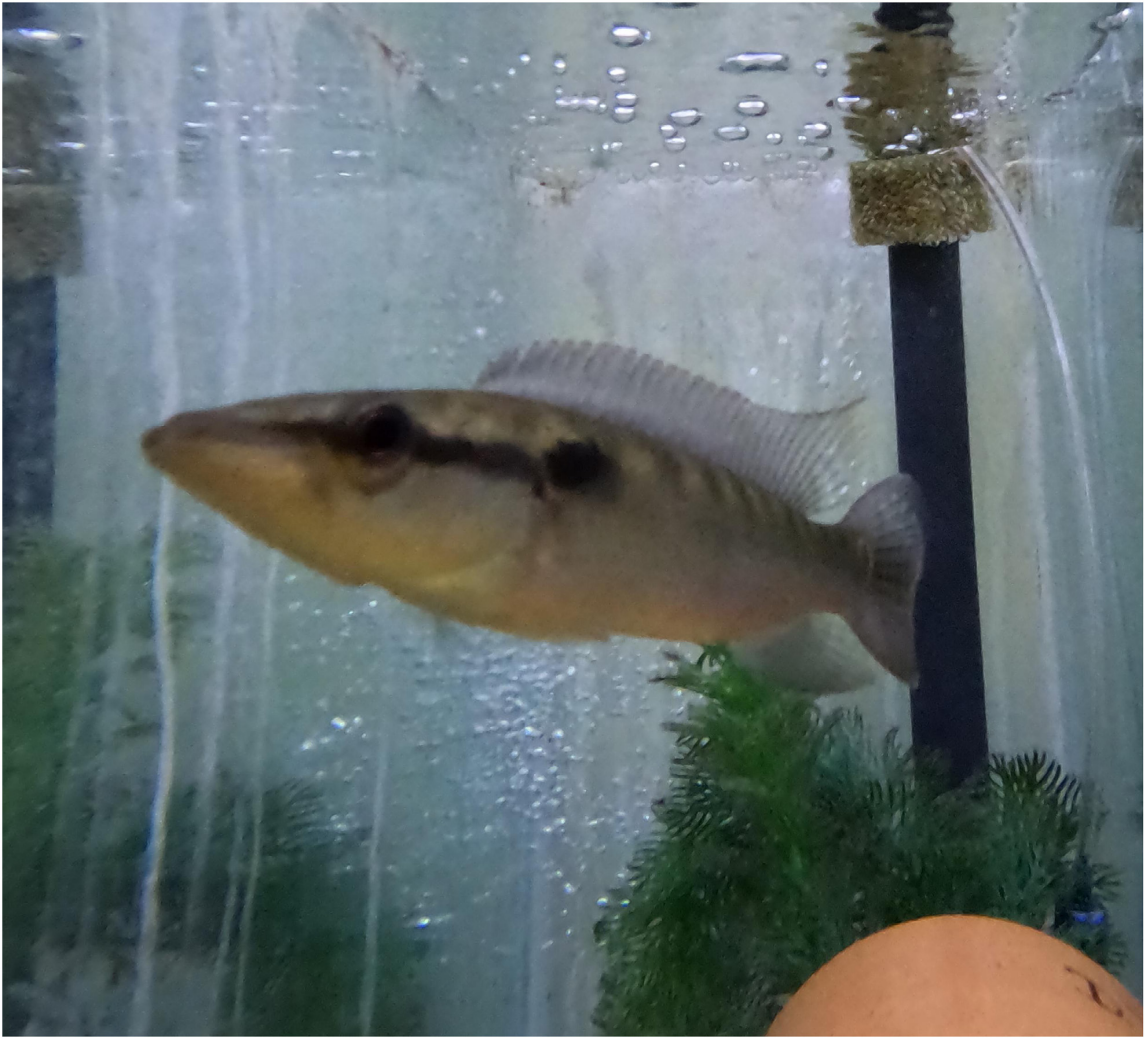
The study species, *Saxatilia proteus*, pictured in their home tank. Credit: Christos Ioannou.

*S. proteus* (mean standard body length ± SD = 9.0 cm ± 1.0 at the time of testing; Figure 1) were reared at the University of Bristol, having been acquired from a commercial aquarium supplier (The Aquatic Store in Bristol); the fish were originally wild caught from Columbia. At this size, we were not able to reliably determine the sex of each individual. The 30 subadult (2‐year‐old) fish were housed individually in 45 L tanks (*L* × *W* × *H* = 70 × 20 × 35 cm) enriched with sand and small stone substrate, plastic tube refuges and artificial foliage. Before the experiment, the water temperature was maintained at 25.8°C ± 0.3 SD, and the light regime followed a 12‐h light–dark cycle. For 3 weeks before testing started, subjects were exclusively fed 2 mL of defrosted BCUK Aquatics Krill Pacifica once a day, which was used throughout the experimental trials because of its consistent sinking behaviour when introduced to the tanks. Opaque white plastic dividers were placed between and behind each tank to eliminate visual cues between subjects, and hence any influence between individuals during the trials. During the trials, each tank had independent filtration provided by a Real Aquatics SF‐101 internal sponge filter. The refuges, foliage and sponge filters were placed in the same configuration in each tank at the rear to ensure they would not obscure feeding during the filming of trials. Weekly water testing for nitrite, nitrate, and ammonia was conducted, in addition to 20% water changes on Mondays. Levels of nitrite and ammonia > 0 ppm, or nitrate > 20 ppm, prompted water changes.

### 2.2 Experimental Treatments and Protocol

Trials were conducted in the subjects' housing tanks to minimise handling and stress, and hence facilitate and standardise feeding. Light intensity was manipulated using LEE 211 0.9ND 3‐Stop Neutral Density Lighting Gel Filters to reduce lux levels within the tanks by approximately 70% (Table 1). The lighting filter sheets were cut to size (70 × 20 cm), with two slits at the rear to accommodate the air and water inflow tubes, enabling the lighting filters to sit flat on top of the tanks. The elevated light treatment comprised of the LED aquarium lighting without the filter (Table 1).

**TABLE 1:** Mean (±SD) temperature and light intensity in tanks during experimental trials measured by HOBO MX2202 loggers. **∆** denotes the difference between control and elevated parameters. The values for the control and elevated temperatures is similar to the minimum and maximum temperatures recorded across sites in the Northern Range mountains of Trinidad that is habitat to *S. frenata* (Zanghi et al. 2024). However, light intensity from this study was typically much higher than in our study, with the minimum average light intensity recorded in the field being 111.6 lx.

	Temperature (°C)	Light intensity (lx)
Control	25.8 (±0.3)	45.5 (±1.9)
Elevated	29.1 (±0.7)	151.6 (±38.3)
**∆**	3.3 (±0.5)	106.1 (±36.4)

**FIGURE A1:**
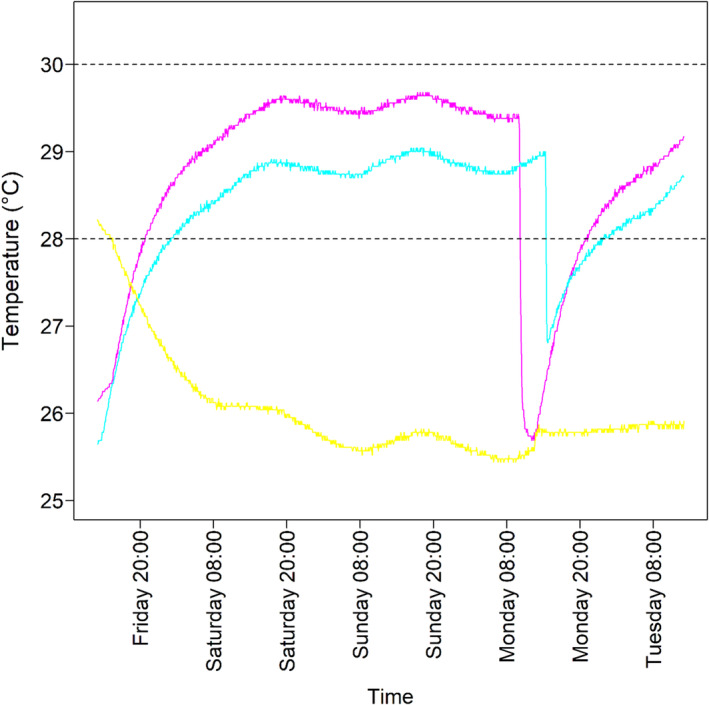
Water heating and cooling profiles for tanks 9 (magenta), 22 (cyan) and 28 (yellow) during the transition from week 1 to week 2 of trials. Temperature readings were captured by HOBO MX2202 loggers at 5‐min intervals. The black dashed lines at 28°C and 30°C represent the lower and upper thresholds for elevated temperature treatments. The transition from week 1 to week 2 treatments occurs on Friday, with trials commencing on Tuesday. The sharp temperature decline on Monday was due to the activation of the inflow system for water changes. Within 24 h, the heaters in tanks 9 and 22 reached the elevated temperature range in preparation for the trials, while tank 28 remained at the control temperature.

AllPondSolutions and HIDOM 25W aquarium heaters were positioned uniformly towards the rear of every tank; water temperature was manipulated by switching each of these heaters on (for the elevated temperature) or off (to maintain the initial housing temperature; Table 1). Each heater had a thermostat that was set to 29°C at the start of the experiment to ensure that minimal interference with the tanks would be necessary once the experiment started. 25 W heaters were used in preference of greater capacity heaters so that the water temperature increased gradually to allow the fish to acclimate and not induce stress (Figure A1). The maximum temperature was limited to 30°C as this was similar to the highest recorded water temperature in the study of Zanghi et al. (2024). The average water temperature increase of 3.3°C in our study reflects projections for river warming over roughly the next 70 years (Liu et al. 2020).

Three HOBO Pendant MX2202 Waterproof Temperature/Light data loggers were used to record lux level and temperature at 5‐min intervals throughout the experiment and were moved between tanks at the end of each testing day. In tanks assigned to the elevated temperature treatment and without a HOBO logger, the temperature was measured using an aquarium thermometer to confirm that the water was at the correct temperature prior to each trial. Experimental trials were conducted between 11 am and 4 pm, Tuesday to Friday over February and March 2024 for four weeks. As the fish were not fed on Saturdays and Sundays as part of their routine husbandry, they were fed on Mondays and experimental trials were only conducted Tuesday to Friday to standardise hunger across the testing days.

The four treatments consisted of a control, an elevated light treatment, an elevated temperature treatment and an interaction treatment where both light and temperature were elevated. The fully factorial design allowed for testing the effects of temperature and light intensity both independently and in combination. During testing, each individual experienced each of the four treatments once for 1 week by manipulating the temperature and light intensity in their home tank. Each tank was part of a block of 6 adjacent tanks; the order of the treatments for each tank was randomised, on the condition that each of the four treatments appeared at least once but not more than twice in that block of 6 tanks in a given week. On each day of testing, the order in which the blocks of 6 tanks were tested was randomised, as was the order of testing of the tanks within each block.

On the Friday of the week prior to the start of experimental trials, treatments for the first week were set up, involving the switching on of heaters and installation of light filters depending on which treatment each tank was assigned to, thus allowing the subjects time to acclimate to their respective treatments for three days before testing (with the exception of a short drop in temperature in the elevated temperature treatment during water changes; Figure A1). Water testing was conducted each Monday morning in addition to water changes, ensuring a full 24‐h period elapsed before testing commenced the following day to allow the water temperature to reach the required temperature after the water change (Figure A1). After testing was completed on Fridays, the next treatment was set up for each tank for the following week.

Before the start of trials each day, 60 g of BCUK Aquatics Krill Pacifica was defrosted in 20 mL of filtered water. The trials were filmed using a Logitech C920 HD Pro Webcam mounted to a Manfrotto camera bracket and clamp. The camera was positioned centrally, facing the narrower vertical wall of the tank (i.e., the wall of dimensions 20 cm wide × 35 cm high), approximately 40 cm in front of the bottom of the tank, angled at 40° upwards to be able to view the entire water column. Video recording was via QuickTime Player (version 10.5) at 1280 × 720 resolution and 30 frames per second. Recording was begun and 2 mL of krill was injected using a 5 mL plastic syringe into the tank through a circular hole in the plastic lid of each tank (the hole was 2 cm in diameter and its centre 4 cm from the front of the tank, positioned centrally along the tank's width). Video 1 demonstrates the beginning of an experimental trial. Feeding was recorded for 5 min from the addition of the food. The camera was then moved to the next tank in the testing order and the trial procedure repeated. A total of 480 trials were conducted; of these, data from 10 trials was missing due to malfunctioning of the recording software. In instances where the heaters malfunctioned, these trials were included as additional replicates for the non‐elevated temperature treatments; this occurred in 9 trials across the duration of the experiment. After testing on the final day, each fish was caught in a net and their standard body length was measured using callipers.

**VIDEO 1:** The first 30 s of an example trial. Video content can be viewed at https://onlinelibrary.wiley.com/doi/10.1002/ece3.70540

### 2.3 Data Processing

Video recordings were analysed using the event‐logging software BORIS (Friard and Gamba 2016). *Crenicichla* make exaggerated jaw movements during feeding (Martinez et al. 2018), and this strike action was recorded as a point behaviour in BORIS. Strikes were used as a measure of feeding motivation rather than food consumption as the food items were not always visible to the experimenter in the video footage. The time in the video that each strike occurred within the 3 min after the food was added was recorded. A single experimenter logged the strikes in BORIS to avoid inter‐experimenter variability. Four response variables were then calculated: the latency to the first strike (seconds), the latency for the 10th strike (seconds), the number of strikes in 60 s, and the number of strikes in 180 s. The latency to the first strike was defined as the time from the introduction of the food into the tank until the first strike was made. Similarly, the latency for the 10th strike was measured from when the food was added to when the 10th strike occurred. For the number of strikes within 60 and 180 s, the time interval (60 or 180 s) started from the time of the first strike. In one trial, no strikes were made in 180 s, and in 5 other trials, less than 10 strikes were made; the total sample size for each response variable is given in the legends of the Tables 2, 3, A1 and A2.

**TABLE 2:** ΔAICc (difference in the Akaike information criterion, corrected for small sample sizes, between the model and the most likely model) model comparisons to determine which explanatory variables and the random effect of subject ID affected the latency to the first strike (natural logarithm transformed). The model with all main effects only is in bold as this is the point of comparison to other models. The df is the number of parameters that are estimated in each model. SBL is standard body length. *N* = 469 trials.

Response variable: log(Latency to 1st strike (s))	ΔAICc	df
Without light	0	8
Without standard body length	0.1	8
Without temperature	0.3	8
Without week of testing	0.6	8
Without day of testing	0.6	8
**All main effects**	**2**	**9**
Without subject ID	2.4	8
Temperature × light	4	10
Without testing order within the day	6.4	8

**TABLE 3:** ΔAICc model comparisons to determine which explanatory variables and the random effect of subject ID affected the number of strikes in the 60 s after the first strike. See Table 2's legend for further details. *N* = 470 trials.

Response variable: Number of strikes in 60 s	ΔAICc	df
Without temperature	0	7
Without standard body length	0.2	7
Without week of testing	0.2	7
Without day of testing	0.2	7
**All main effects**	**2**	**8**
Without light	2.5	7
Without testing order within the day	3.7	7
Temperature × light	4.1	9
Without subject ID	157.1	7

### 2.4 Statistical Analysis

R version 4.3.3 was used to conduct the statistical analyses. Using the lmer and glmer functions in the lme4 package (Bates et al. 2015), each response variable (i.e., the latency to first strike, latency for the 10th strike, number of strikes in 60s and number of strikes in 180 s) was analysed separately in a (generalised) linear mixed model ((G)LMM). The temperature treatment (control or elevated) and light treatment (control or elevated) were included as fixed factors, as well as the interaction term between them. Testing order within the day (1–30), day within the week (1–4), week number (1–4), and standard body length were included as main‐effect only covariates in the models; these were all transformed using the scale function in R (i.e. mean centred with mean = 0 and standard deviation = 1) to avoid problems with model fitting. Consistent individual variation between the subjects was modelled using a random effect of subject identity. The DHARMa package (Hartig 2019) was used to check the assumptions of the models, specifically the distribution of residuals (confirmed using Q–Q plots) and the relationship between the residuals and the fitted values. As the latency to the first strike and the latency for the 10th strike did not meet the assumptions of a linear model, they were log‐transformed and 1/square root transformed before analysis, respectively; these transformations were determined using Box‐Cox transformations (Box and Cox 1964), and the models met the assumptions after these transformations. The number of strikes in 60 s and 180 s were modelled with a Poisson distribution as they are count data, and met the assumptions tested by the DHARMa package, including that the dispersion parameter was approximately equal to 1.

To determine whether the light intensity × temperature interaction, each of the main effects, and the random effect of subject ID were important in predicting feeding motivation, for each response variable we constructed a model that included all main effects and the interaction term, a model that included all the main effects only, and models that removed only one of the main effects, or the random effect, in turn from the all main‐effects model (Tables 2, 3, A1 and A2). Model comparisons were carried out with the Akaike information criterion, corrected for small sample sizes (AICc), using the ICtab function from the bbmle package (Bolker and R Development Core Team 2017). If the model with a removed term is more likely (lower AICc), this suggests that the removed term is not important to include in the model, i.e., does not explain an adequate amount of the variation in the response variable. The contrary is true if removing a term makes the model less likely (higher AICc). A difference of > 2 AICc units between models can be considered strong support for the model with the lower AICc (Burnham and Anderson 2002). To quantify interindividual differences in feeding motivation, estimates of repeatabilities and their 95% confidence intervals (CIs) were obtained using the rpt function from the rptR package (Stoffel, Nakagawa, and Schielzeth 2017) for each of the four response variables, using the same model families (i.e., Gaussian for latencies and Poisson for the number of strikes) as used in the models in the AICc comparisons.

### 2.5 Ethical Note

The study was approved by the University of Bristol Animal Welfare and Ethical Review Body (UIN/23/074). Elevated temperature treatments were limited to 30°C and increased gradually between treatments (Figure A1) to minimise physiological stress. Water testing and changes were conducted weekly to ensure high water quality. After the experiment, the fish remained housed in the University of Bristol's research facility to be used in future experiments. No fish died or showed signs of ill health during the experimental period, and all subjects were still in good health as of October 2024.

## 3 Results

**FIGURE 2:**
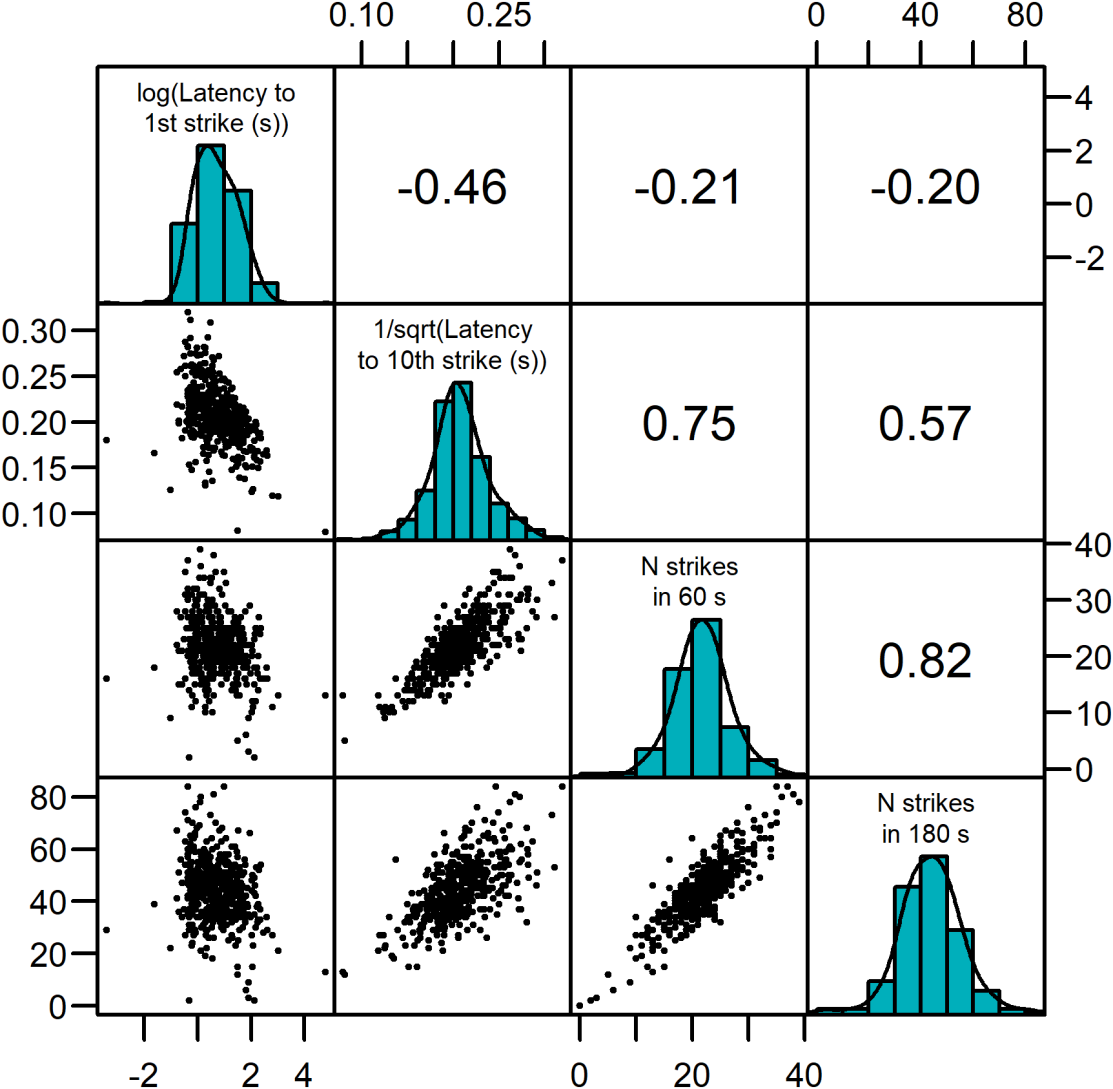
Correlations between the measures of feeding motivation (i.e., latency to the first strike, latency to the 10th strike, number of strikes in the 60s, and number of strikes in 180 s). The values in the top right section of the plot are Spearman's rank correlation coefficients. Note that with the 1/square root transformation for the latency to the 10th strike, larger values indicate shorter latencies.

The four measures of feeding motivation varied in the extent that they were correlated with one another (Figure 2). The latency to the 10th strike and the number of strikes in both 60 s and 180 s were more strongly correlated than any of these were correlated with the latency to the first strike, although there was a moderate correlation between the latency to the first and 10th strikes.

**FIGURE 3:**
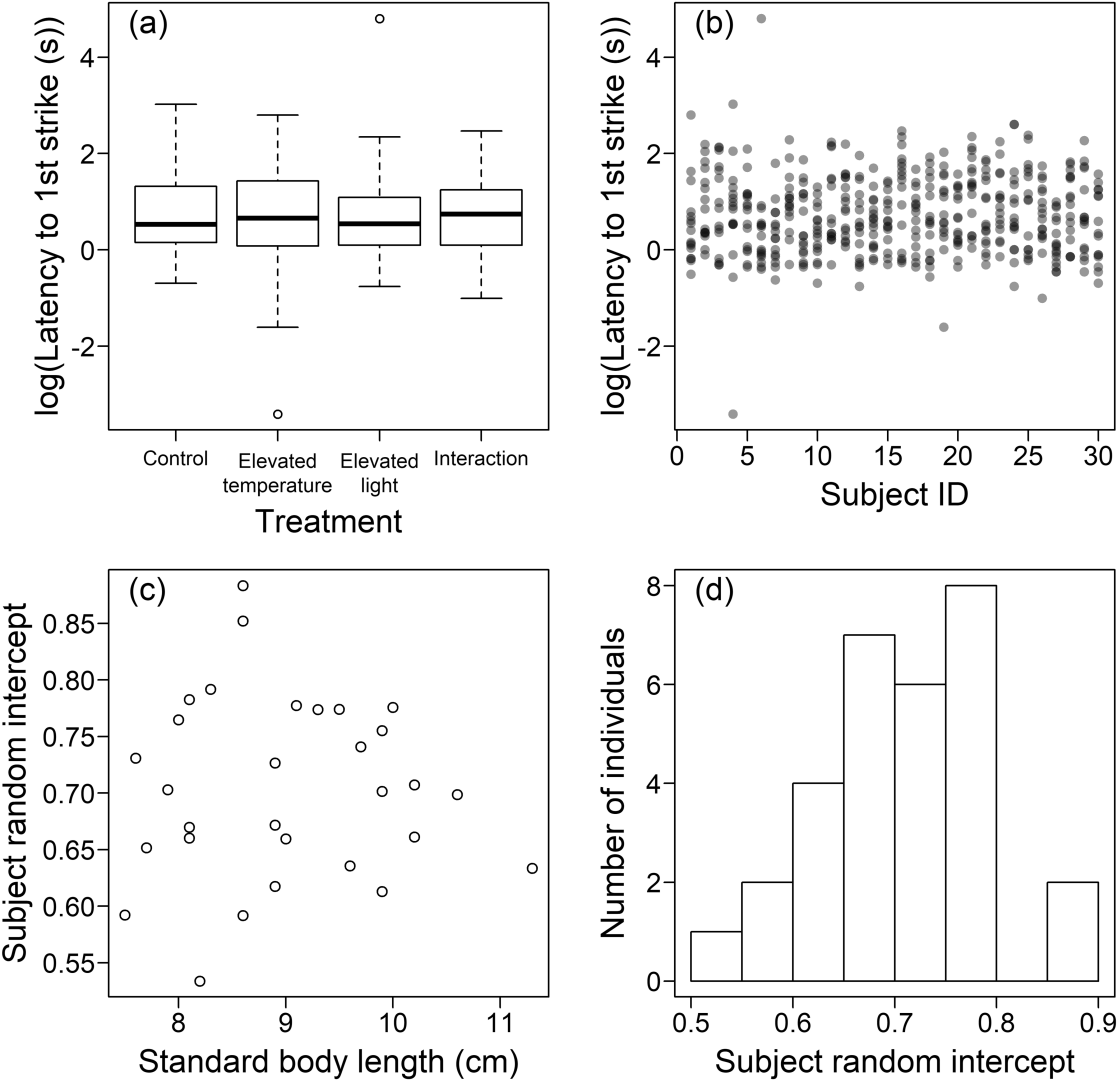
The latency to the first strike as a measure of feeding motivation. (a) Shows how the latency to the first strike was affected by the manipulated environmental variables, and (b) how it varied between the different individual fish. Using the intercept fitted for the random effects of subject ID, (c) shows how differences between the fish in the latency to the first strike varied with body size, and (d) shows the distribution of these random intercepts. In (a), the box length denotes the interquartile range, and the median value is represented by the horizontal black line inside the boxes. The vertical black dashed lines indicate data points within 1.5 times the interquartile range above and below the upper (75%) and lower quartiles (25%). The circles represent outliers.

**FIGURE 4:**
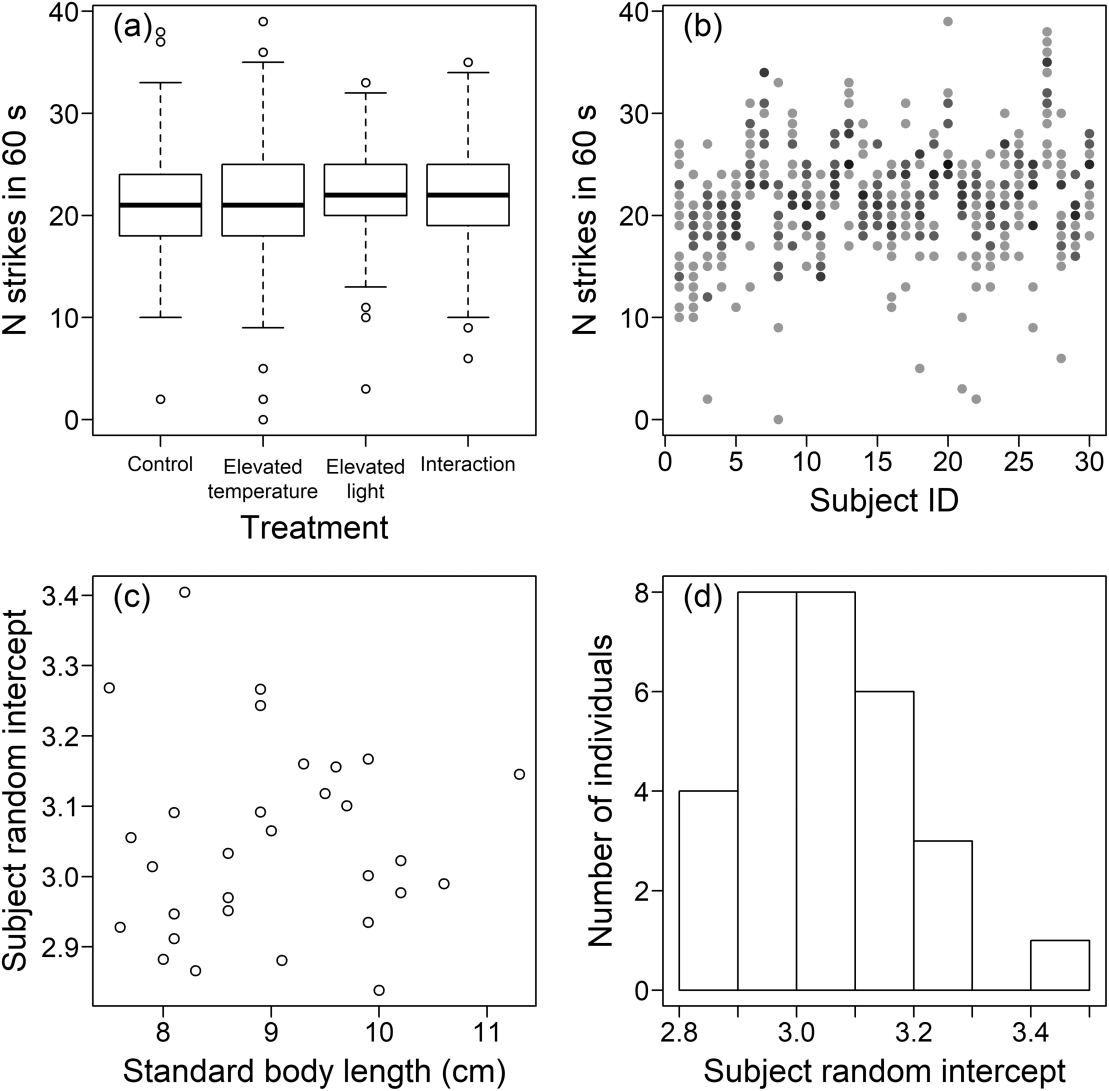
The number of strikes in the first minute as a measure of feeding motivation. Plotting as in Figure 3.

**FIGURE A2:**
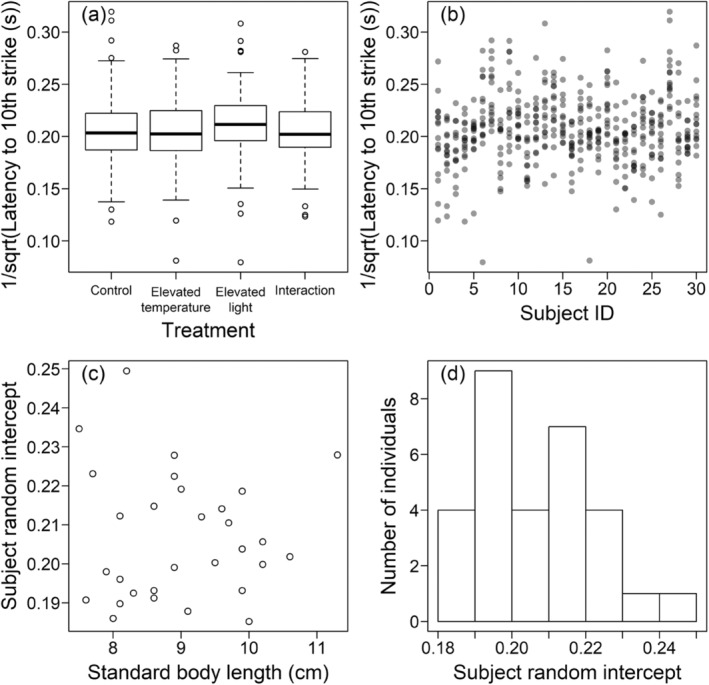
The latency to the 10th strike as a measure of feeding motivation. Plotting as in Figure 2.

**FIGURE A3:**
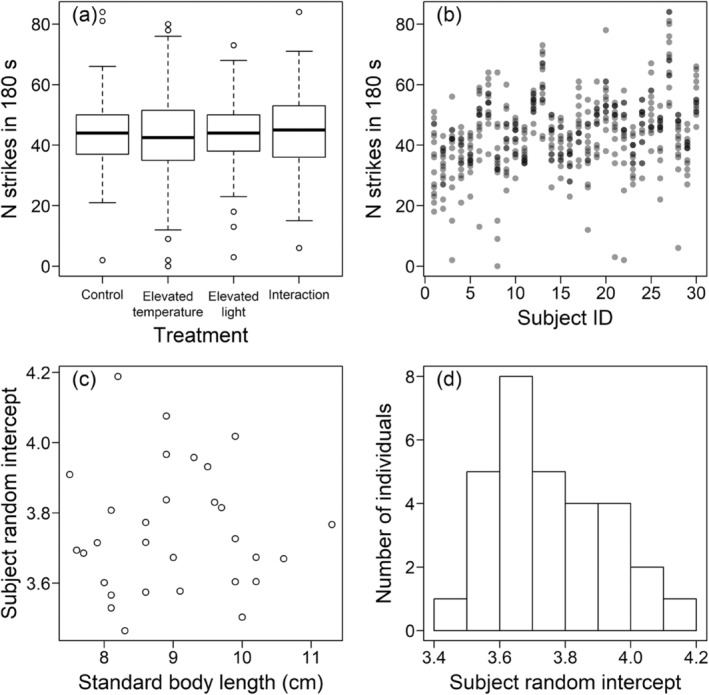
The number of strikes in the first 3 min as a measure of feeding motivation. Plotting as in Figure 2.

For all four measures of feeding motivation, including the temperature × light treatment interaction made the models less likely, i.e., the models with the interaction had a higher AICc (Tables 2, 3, A1 and A2, Figures 3a, 4a, A2a and A3a). Removing the temperature treatment main effect from the all main‐effects models made the models more likely, providing evidence that the temperature treatment as a main effect did not affect any of the measures of feeding motivation. Similarly, removing the light treatment main effect improved the model likelihood for the latency to the first and 10th strikes (Tables 2 and A1). While removing the light treatment term worsened the likelihood of the number of strikes in 60 and 180 s, this was only by 0.5 and 0.8 AICc units, respectively (Tables 3 and A2), so there was no strong evidence that including this variable in the all main‐effects model improved the model likelihood.

Of the covariates, removing standard body length from the all main‐effects models improved the likelihood for all four measures of feeding motivation (Tables 2, 3, A1 and A2), suggesting no association between body size and feeding motivation. Removing the day of testing within the week (1–4) improved the model likelihoods, or, for the latency to the 10th strike, reduced the likelihood by only 0.5 AICc units (Table A1). The model comparisons for the week of testing (1–4) showed that it was not important to include in the models predicting the latency for the first strike or the number of strikes in 60 s, but there was strong evidence that removing this term reduced the model likelihood for the latency to the 10th strike (ΔAICc = 2.6) and number of strikes in 180 s (ΔAICc = 13.2). While the latency to the 10th strike increased over the weeks of testing (estimated slope: −0.00277, standard error: 0.00127; note that the slope is negative as this latency was 1/sqrt transformed), suggesting a reduced feeding motivation, in contrast the number of strikes in 180 s increased as the weeks progressed (estimated slope: 0.0273, standard error: 0.00699).

There was evidence from all four measures that the testing order within the test days (1–30) influenced the feeding motivation of the fish (Tables 2, 3, A1 and A2). For the number of strikes in 60 s, there was moderate evidence for this (the ΔAICc between the all main‐effects model and the model without the testing order was 1.7; Table 2), while there was strong evidence for the other three measures of feeding motivation (ΔAICc > 2.5). Feeding motivation decreased as the testing progressed on each day: the latency to the first strike increased (estimated slope: 0.0950, standard error: 0.0371), and to the 10th strike also increased (estimated slope: −0.00517, standard error: 0.00134; note that the slope is negative as this latency was 1/sqrt transformed). The number of strikes in 60 (estimated slope: −0.0203, standard error: 0.0105) and 180 (estimated slope: −0.0160, standard error: 0.00742) seconds decreased.

For the latency to the first strike, removing the random effect of subject identity reduced the model likelihood by only 0.4 AICc units (Table 2, Figure 3b). The individual identity of the fish was, however, important to include in predicting the number of strikes within the first 60 s (Table 3). Figure 4b demonstrates the inter‐individual variation in the number of strikes within the first 60 s. As the standard body length was not important to include in the model, the consistent inter‐individual variation was not due to differences in body size, and Figure 4c demonstrates no association between body length and the individual‐level intercepts fitted from the all main‐effects model. The distribution of the individual‐level intercepts is unimodal (Figure 4d), suggesting that the consistent inter‐individual variation was not driven by differences between sexes, as strong sex differences would be expected to generate a bimodal distribution (i.e., a mode for each sex); however, without sexing the fish we cannot rule out more subtle sex differences increasing the extent of consistent inter‐individual variation. Consistent with the correlations between the measures of feeding motivation, the importance of inter‐individual differences was replicated with the number of strikes within 180 s and the latency to the 10th strike (Tables A1 and A2, Figures A2 and A3).

As the AICc model comparisons demonstrated strong consistent variation between the individual fish that could not be accounted for by differences in body size, and which are unlikely have been due to sex differences, we calculated repeatability estimates for the measures of feeding motivation. Consistent with the model comparisons, the repeatability estimate was low and the 95% confidence intervals included zero for the latency to the first strike (Table 4). However, the other three measures of feeding motivation demonstrated moderate repeatability estimates with confidence intervals that did not include zero (Table 4).

**TABLE 4:** Repeatability and variance estimates for the subject identity random effect. The between and residual variance are from the all main‐effects models, and includes the standard deviation (SD) in brackets. The residual variance could not be calculated from the number of strikes due to the Poisson distribution specified in the GLMMs.

Response variable	*R*	Lower 95% CI	Upper 95% CI	Between individual variance (SD)	Residual variance (SD)
log(Latency to 1st strike (s))	0.030	0.000	0.082	0.019 (0.138)	0.617 (0.785)
Number of strikes in 60 s	0.307	0.169	0.416	0.021 (0.143)	NA
1/sqrt(Latency to 10th strike (s))	0.260	0.147	0.382	0.000291 (0.0171)	0.000749 (0.0274)
Number of strikes in 180 s	0.412	0.237	0.526	0.0321 (0.179)	NA

## 4 Discussion

Although temperature and light intensity have been confirmed to impact feeding in other species (Domenici et al. 2019; Fleming and Bateman 2018; Richmond, Hrabik, and Mensinger 2004; Volkoff and Peter 2006), within the ranges tested in our study we found no effect of these stressors on the feeding motivation of *S. proteus* . There was, however, evidence of consistent inter‐individual differences, i.e., personality variation, in this population of *S. proteus* in their motivation to feed. Thus, the composition of a predator population is more likely to impact prey, with potentially knock‐on effects for the ecosystem via lethal and non‐lethal effects (Binckley and Resetarits 2003; Resetarits and Pintar 2016; Rudolf 2012), than changes in temperature and light intensity that would be expected to occur through habitat change such as the removal of canopy cover over freshwater streams from deforestation.

The composition of a predator population with respect to their feeding motivation will vary depending on both natural and anthropogenic factors. Although our study only used subadult individuals, the study of Szopa‐Comley et al. (2020) of wild *S. frenata* studied in situ also showed consistent inter‐individual variation in feeding motivation, and this was likely to have included sexually mature individuals. For mesopredators such as pike cichlids, populations can become more risk‐averse with an increased perception of predation risk, including a decreased feeding rate due to the trade‐off between predation risk and foraging (Verdolin 2006). In addition to this non‐lethal effect, predation and other risks can selectively remove less risk‐averse individuals from a population through direct mortality (Bell and Sih 2007; Dhellemmes et al. 2021), although this is not always the case (Balaban‐Feld et al. 2019; Dhellemmes et al. 2021). Harvesting by humans, either for recreation or commercially, is frequently non‐random with respect to the risk‐taking tendency of the individuals harvested, thus shifting the average risk‐taking tendency in the population, usually toward shyer, more timid behavioural types (Arlinghaus et al. 2017; Biro and Post 2008; Monk et al. 2021). Thus, harvesting of wild populations can act as an anthropogenic stressor indirectly impacting prey consumption by altering the behavioural composition of predator populations.

Increased temperature and light intensity are not the only effects of deforestation around freshwaters, which includes alteration of hydrological and water chemistry parameters (Castello et al. 2013; Ríos‐Villamizar et al. 2017). Increased run‐off and sedimentation caused by deforestation contributes to increased water turbidity, which declines the rate of predation by visual predators (Ehlman, Torresdal, and Fraser 2020; Lunt and Smee 2015). However, Zanghi, Munro, and Ioannou (2023) found that in turbid, warm water, guppies *Poecilia reticulata* (prey of the pike cichlid *S. frenata*) reduced shoaling and increased proximity to another predatory fish, the blue acara ( *A. pulcher* ), suggesting that these conditions could be advantageous to predators by reducing prey anti‐predator behaviour. Increased light penetration from reduced canopy cover from deforestation could be counteracted by more frequent and severe incidences of elevated turbidity, while the elevated temperature could increase the energetic demand of predators. Habitat change, driven by the single human activity of deforestation, can thus alter multiple environmental parameters that can potentially impact predator–prey interactions in freshwater ecosystems.

By conducting the trials before the fish were fed each day, our study was designed to infer feeding rates when motivation to feed would be high. This may have reduced the extent of inter‐individual variation observed in our study compared to a situation where all fish were partially satiated, as individuals typically less motivated to feed may reduce their feeding more rapidly as they feed, increasing the differences between less and more motivated fish. However, over longer time scales, piscivore fish become fully satiated when prey are abundant, which is a likely driver for type II and type III functional responses being common in piscivores under natural conditions (Moustahfid et al. 2010). When prey are abundant, individuals with a greater motivation to feed will satiate earlier, reducing their consumption of prey, and reducing the difference in feeding rates compared to less motivated predators that are less sated. Such a state‐behaviour feedback (Sih et al. 2015) will reduce inter‐individual variation in feeding. Research on three‐spined sticklebacks ( *G. aculeatus* ) has demonstrated consistency in individual differences was reduced in a foraging context but maintained in control trials where no food was present (MacGregor, Cottage, and Ioannou 2021), although the opportunity to forage tended to make individuals less predictable rather than reduce variation between individuals (also see Brand et al. 2021; Mitchell and Biro 2017). Further research in this area is needed to determine the extent of consistent inter‐individual variation between predators at different prey densities. Another factor which may suppress consistent differences between individuals is conformity, where individuals become more similar in their behaviour due to signals or cues from one another (Ioannou and Laskowski 2023). Although adult pike cichlids are not found in social groups other than reproductive pairs, multiple individuals can occupy the same pools and be within visual range of one another (Szopa‐Comley et al. 2020; Zanghi et al. 2024), and social information can be used in a foraging context even in fish species that do not live in groups (Webster and Laland 2017). The extent to which consistent inter‐individual variation within a predator population is supressed by social information leading to conformity deserves further study.

The latency to the first strike was not consistently different between individual *S. proteus* , and this variable was not strongly correlated with the other three variables used to quantify feeding motivation (the latency to the 10th strike, and the food consumed in the first minute and first 3 min), which were all correlated with one another. While the latency to the first strike has been used as an indicator of feeding motivation in fish previously (Volpato et al. 2013), in our study this variable may have been sensitive to the location and orientation of subjects within the tank when the food was introduced, contributing unaccounted variation in the statistical models predicting the latency to the first strike. The latency to the first strike has been shown to be correlated to refuge use and thigomotaxis in separate trials among individuals in other fish species (e.g., eastern mosquitofish, *Gambusia holbrooki* ) (Brand et al. 2023). If variability in space use in our study explained the lack of repeatability in the latency to the first strike, it suggests that space use itself was not repeatable between individual *S. proteus* . This may be because the feeding trials in our study were conducted in the fish's home tanks to reduce stress by being a familiar environment and to avoid handling before the trials, and hence facilitate feeding. With a different tank arrangement that would allow filming from multiple perspectives, the fish's location and orientation within the tank could be quantified and included as covariates in the statistical models (as in MacGregor, Herbert‐Read, and Ioannou 2020). The use of pose tracking software (e.g., Pereira et al. 2022) would also allow kinematic measurements of the strikes. Alternatively, when investigating the effect of temperature on feeding motivation in Pacific halibut *Hippoglossus stenolepis* , Stoner, Ottmar, and Hurst (2006) arranged multiple vertical feeding tubes in each tank for food introduction. This setup allowed food to emerge at the greatest distance from the test subject, standardising subject positioning within tanks and minimising potential confounding effects on latency measurements. In our study, the variable location and orientation of the subjects within the test tank when the food was first introduced is likely to have had a reduced contribution to the variability in strikes after the first strike, explaining why we were able to detect consistent inter‐individual variation in the latency to the 10th strike, and the food consumed in the first minute and first 3 min.

Overall, our study suggests that feeding motivation in pike cichlids is robust to relatively short‐term exposure to changes in temperature and light intensity that is likely to be associated with removal of canopy cover from deforestation adjacent to freshwater streams. Instead, individuals of *S. proteus* show consistent differences in their feeding motivation, and these differences were unrelated to the body size of the individuals. A similar trend of consistent inter‐individual variation in feeding that was independent of body size has been also demonstrated in another pike cichlid, *S. frenata*, under field conditions (Szopa‐Comley et al. 2020), and in northern pike *E. lucius* (Nyqvist et al. 2012). Despite the prevalence of consistent inter‐individual differences in predator populations, the ecological impacts of these differences have yet to be extensively explored, which are likely to be varied. For example, individual predators posing a greater risk to prey are likely to have different spatial distributions to less dangerous individuals, creating risk landscapes for their prey (Dammhahn, Lange, and Eccard 2022; Steinhoff et al. 2020). Predator–prey interactions in freshwater aquatic systems, which can be studied under both laboratory and field conditions and are amenable to experimental manipulation as well as observation, are particularly promising for future studies in this area.

## Author Contributions

**Lucy J. Brown:** conceptualization (supporting), data curation (lead), formal analysis (supporting), investigation (lead), methodology (equal), visualization (supporting), writing – original draft (lead), writing – review and editing (supporting). **Christos C. Ioannou:** conceptualization (lead), formal analysis (lead), funding acquisition (lead), methodology (equal), resources (lead), supervision (lead), visualization (lead), writing – review and editing (lead).

## Conflicts of Interest

The authors declare no conflicts of interest.

## Supporting information


Data S1

## Data Availability

The data and R code for the analyses are provided as Supporting Information.

## Appendix 1

**TABLE A1:** ΔAICc model comparisons to determine which explanatory variables and the random effect of subject ID affected the latency to the 10th strike. See Table's legend for further details. *N* = 464 trials.

Response variable: 1/sqrt(Latency to 10th strike (s))	ΔAICc	df
Without standard body length	0	8
Without temperature	1.5	8
Without light	1.9	8
**All main effects**	**2**	**9**
Without day of testing	2.5	8
Temperature × light	3.3	10
Without week of testing	4.6	8
Without testing order within the day	14.5	8
Without subject ID	93.1	8

**TABLE A2:** ΔAICc model comparisons to determine which explanatory variables and the random effect of subject ID affected the number of strikes in the 180 s after the first strike. See Table 2's legend for further details. *N* = 470 trials.

Response variable: number of strikes in 180 s	ΔAICc	df
Without temperature	0	7
Without day of testing	0.1	7
Without standard body length	0.2	7
**All main effects**	**1.7**	**8**
Temperature × light	2	9
Without light	2.5	7
Without testing order within the day	4.2	7
Without week of testing	14.9	7
Without subject ID	609.8	7
